# Higher Mortality in Men Compared with Women following Distal Radius Fracture in Population Aged 50 Years or Above: Are Common Distal Radius Fracture Classifications Useful in Predicting Mortality?

**DOI:** 10.1155/2019/5359204

**Published:** 2019-01-23

**Authors:** Jakub Marchewka, Jacek Głodzik, Wojciech Marchewka, Edward Golec

**Affiliations:** ^1^Department of Physiotherapy, University of Physical Education, Kraków 31-571, Poland; ^2^Department of Orthopedics and Trauma Surgery, 5^th^ Military Hospital, Kraków 30-901, Poland; ^3^Jagiellonian University Medical College, Kraków 31-008, Poland

## Abstract

**Introduction:**

Distal radius fractures (DRF) are one of the most common fractures with growing incidence in developed countries and are a reliable predictor of another osteoporotic fracture. Data concerning DRF mortality are conflicting and vague. Usefulness of common DRF classification systems in predicting mortality is unexplored.

**Methods:**

We identified all patients hospitalized between January 1^st^ 2008 and May 30^th^ 2015 with isolated distal radius fracture, aged 50 y/o or above, in a 1^st^ level trauma center in Poland. Fractures were evaluated according to AO, Frykman, and Fernandez classifications. Mortality ratios and long-term survival analysis with Kaplan-Meier estimator and log-rank tests with univariate and multivariate Cox proportional hazards model were used.

**Results:**

We enrolled 1308 consecutive patients. The average age of the entire cohort was 72.5 ± 12 years. The study group consisted of 256 men (19.6%) with mean age 66 ± 12 y/o and 1052 women (80.4%) with mean age 74 ± 12 y/o. Men were statistically younger at the time of the fracture than women (p<0.0001). After 1-year follow-up the overall study group mortality ratio was 4.5%, being 2.2-fold higher in men compared to women. In long-term survival analysis, excess men mortality remained significant. Factors associated with higher mortality at any point of the study were age (HR: 1.08, 95%CI: 1.07-1.10, p<0.000001), male sex (HR: 1.92, 95%CI: 1.34-2.77; p<0.001), AO type A (HR: 1.64 95%CI 1.19-2.25, p<0.01), and Frykman type I (HR: 2.12 95%CI: 1.36-3.29, p<0.001).

**Conclusion:**

Distal radius fractures are connected with premature mortality. Men have higher mortality compared with women following distal radius fracture in population aged 50 years or above. Simple extra articular fractures classified as AO type A or Frykman type I may be predictors of higher mortality in DRF cohort.

## 1. Introduction

Distal radius fractures (DRF) are one of the most common types of fractures [1]. In the United States alone the annual incidence is estimated at 16.2 DRF fractures per 10 000 population [2], which amounts to over 650 000 fractures per year [1]. They generate huge socioeconomic costs associated with disability, cessation or reduction of work productivity, and functional impairment [3, 4]. In the European Union, the annual cost of fragility fractures is estimated at about 37 billion euros, with predicted increase of 25% in the years from 2010 to 2025 [5]. The majority of distal radius fractures occur in adult population, with underlying osteoporosis [6]. Long-term observations indicate that that the incidence of DRF increases, what is attributed to continuous aging of the population, growing life expectancy, and an epidemic of osteoporosis [7, 8]. Lifetime risk of osteoporotic fracture at age 50 is approximately 39.7-53.2% amongst women and 13.1-20,7% in men, or, when taking into account distal radius fractures, 13.3-20.8% amid women and 2.5-4.6% in men accordingly [6]. Distal radius fractures have many possible short- and long-term unfavorable sequelae on an individual's health leading to deterioration of functional and general health status [9].

It is well established that distal radius fractures are a good predictor of osteoporosis [10] and another, often more severe, fragility fracture [11, 12]. The premature mortality following fragility fractures such as hip and vertebral fractures is also well documented and undebatable [13, 14]. However, the results concerning mortality after distal radius fractures are fragmentary and conflicting [9, 11, 15]. Furthermore, the usefulness of common distal radius fracture classifications, used in everyday practice, in predicting mortality is unexplored.

The first aim of this study was to analyze short- and long-term mortality after distal radius fracture in population aged 50 years old or above, compare it with the standard population, and calculate expected years of life lost in the analyzed cohort. Secondly, we aimed to investigate certain risk factors affecting mortality, paying particular attention to AO, Frykman, and Fernandez distal radius fracture classifications results.

## 2. Materials and Methods

### 2.1. Participants

We identified all consecutive patients hospitalized between January 1^st^ 2008 and May 30^th^ 2015 with distal radius fracture in a 1^st^ level trauma center in Cracow, Poland. It was a two-step identification process. Firstly, we recognized all patients with S52.5, S52.6, and S62.8 International Classification of Diseases 10th Revision (ICD10) codes. Secondly, senior orthopedic hand surgeon specialist and resident assessed dually and simultaneously the radiographs in order to confirm the diagnosis. Subsequently, after conformation of fracture, we collected the following data: age, sex, hand side, and residency. The distal radius fracture was classified according to AO, Frykman, and Fernandez classification system. Taking into consideration results of preliminary logistic regression and corresponding data from the literature [15–19], we have appointed inclusion criteria as follows: age ≥ 50 years, isolated distal radius fracture confirmed by X-ray examination in standard PA and lateral view. Multitrauma patients were excluded from the study. The censoring date of follow-up was 31 May 2016.

### 2.2. Mortality and Years of Life Lost

The primary outcome was mortality. Cumulative mortality was assessed 3, 6, and 9 months and 1 year after distal radius fracture and analyzed within sex groups. We calculated crude and standardized mortality ratios (SMRs). SMR is the ratio of the observed to the expected number of deaths in the study population under the assumption that the mortality rates for the study population are the same as those for the general population [20]. SMRs were calculated using age- and sex-specific data obtained from the Polish Central Statistical Office. The data concerning mortality in studied population was provided by Ministry of the Interior and Administration of the Republic of Poland. In order to calculate the number of years of life lost by the studied DRF population in comparison to the years lost by the referential, standard population, we calculated the standard expected years of life lost per living person (SEYLLp) in the studied population index. Life expectancy tables of the European Union ‘old' 15 countries according to the authors of ‘Health statistics – Atlas on mortality in the European Union' were adopted as standard [21]. Standard populations statistics were gathered from Eurostat database.

### 2.3. Statistical Analysis

To establish inclusion criteria, we performed multivariate explanatory logistic regression. Afterwards, to compare the results between genders, we conducted survival analysis with Kaplan-Meier estimator and log-rank tests. We used univariate and multivariate Cox proportional hazards model to calculate hazard ratios (HRs) with 95% confidence intervals. The model was tested for proportional hazards assumptions, after adjustments. To perform standardized mortality ratios (SMRs) analysis, we calculated Mid-P exact test using Miettinen's modification, as described in [22]. The abovementioned Mid-P test was calculated by OpenEpi software [23]. All other calculations were performed using Statistica 12.5 (StatSoft ® Inc. USA). All p-values are two-sided, p <0.05 was considered statistically significant.

### 2.4. Ethical Approval

The study was approved by the Local Chamber of Physicians and Dentists Bioethics Committee in Cracow, approval no: 141/KBL/OIL/2015. All the procedures complied with the Helsinki Declaration.

## 3. Results

We enrolled 1308 consecutive patients with isolated distal radius fracture aged 50 years or above in our single-center, retrospective study. The average age of entire cohort was 72.5 ± 12 years. The study group consisted of 256 men (19.6%) with mean age 66 ± 12 y/o and 1052 women (80.4%) with mean age 74 ± 12 y/o. Men were statistically younger at the time of the fracture than women (p<0.0001). After 1-year follow-up the overall study group mortality ratio was 4.5%. Comparing the two genders, we found that 1-year cumulative mortality ratio was 2.2-fold higher in men than women with distal radius fracture, even though men were approximately 8 years younger than women at the time of the fracture. When study population was additionally subdivided into 50-59, 60-69, and 70+ age groups, even higher 1-year excess mortality between males and females was observed in individuals above 60 y/o, comprising 4.4-fold and 3.5-fold increase in 60-69 and 70+ age group, respectively, as shown in Table 1. Comparison to the age- and sex-matched standard Polish population revealed statistically significant standardized mortality ratios (SMRs) increase in mortality within men group, especially those aged 70 years and more, as presented in Table 1. In our cohort, patients who died were on average 11 years older at the time of the fracture than those who lived (p<0.001).

**Figure 1:**
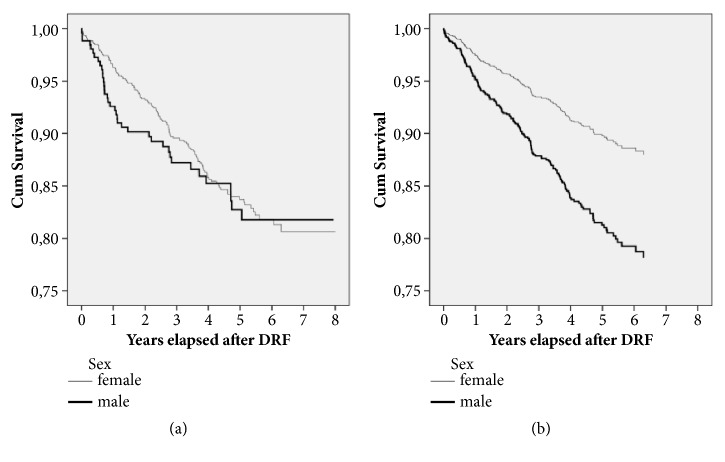
Unadjusted (a) and age-adjusted (b) Kaplan-Meier curves illustrating survival probabilities in studied distal radius fracture cohort divided into sex subpopulations. Normal line: females, bold line: males, DRF: distal radius fracture, cum survival: cumulative survival.

The differences between males and females in cumulative, overall crude mortality after distal radius fractures were statistically significant from the 6^th^ month of fracture (2.7% vs 1.5%, p=0.003) until the 4^th^ year of follow-up. Afterwards the mortalities were comparable, but still higher than those in standard age- and sex-matched population. After controlling for age [24, 25] male mortality was significantly higher than female mortality, as illustrated in Figure 1. In long-term survival analysis, excess male mortality remained significant.

The estimated standard expected years of life lost per living person (SEYLLp) in overall studied distal radius fracture population was 0.95 years. Comparing the two genders, we found that SEYLLp within men was higher than women: 1.6 years versus 0.6 years, respectively.

**Figure 2:**
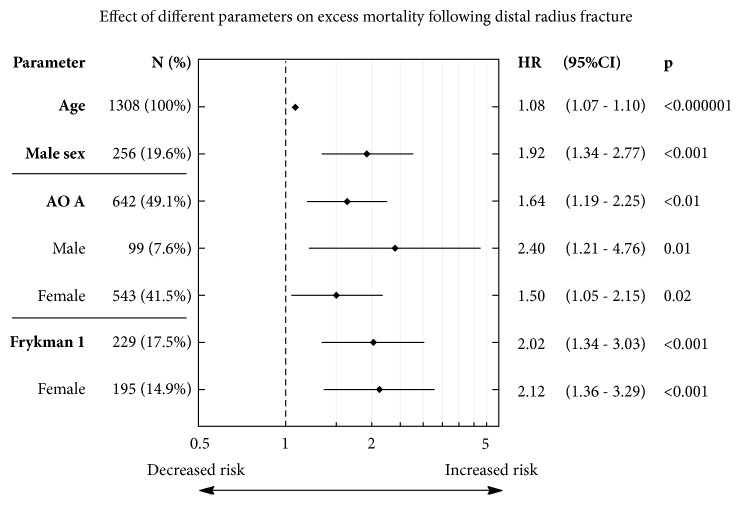
Forrest plot of risk factors for death after distal radius fracture (DRF) in study cohort. The data were obtained from Cox proportional hazards model and presented as hazard ratios (HRs) with 95% confidence intervals (95% CI). On the left side of the graph a subdivision into sex populations was made, if statistically significant. AO A: type A of DRF according to AO foundation.

Further investigation with Cox proportional hazards model analysis confirmed that age is associated with higher risk of mortality (HR: 1.08, 95% CI: 1.07-1.10, p<0.000001). Multivariate Cox regression with adjustment for age indicated that males were almost twice more likely to die than females (HR: 1.92, 95% CI: 1.34-2.77; p<0.001) at any point of the study. Furthermore, patients with type A distal radius fracture, according to AO classification, were more likely to die than those with AO B and C altogether. After dividing AO cohorts into sex subgroups, the hazard ratio was even higher in male subpopulation. Patients with Frykman I DRF diagnosis were more likely to die than those with Frykman II-VIII accordingly. We have not observed significant differences in mortality between 1-5 Fernandez DRF classification system subgroups. Neither left nor right hand or handedness of broken radius was connected with estimated survival. Statistically significant factors affecting mortality in distal radius fracture 50+ cohort were presented in Figure 2.

## 4. Discussion

We found that overall mortality in distal radius fracture cohort was higher than in the standard population. Overall 1-year mortality in our study group, 4.5 %, was greater than that observed by Øyen [15] and Endres [26] (3.4 % and 3% accordingly), but lower than the 6% reported by Johnell [27]. Comparing the study cohort with the background and standard age- and sex-matched population, we found significant decrease in men survival, which is in accordance with Rozental [28] and Shortt [9] observations. Contrarily Øyen [15], Endres [26], Shauvier [29], and Johnell [6, 27] found no significant differences in mortality ratios between DRF and similar general population. The abovementioned dissimilarities may be linked with the methodological issues concerning inclusion criteria (isolated vs nonisolated fracture) or public health system alterations over time, including better osteoporosis and fall prevention measures. Interestingly, our findings on reduced life expectancy after DRF are parallel to those of hip and vertebral fractures, whilst distal radius fractures are harbingers for other osteoporotic fractures [30]. Bliuc et al. reported 5-year accumulative incidence of subsequent fracture after an initial osteoporotic fracture as 24% in females and 20% in males [31]. We think that distal radius fracture mortality is unlikely to be the result of acute injury per se. It may be explained by limited mobility, reduced activities of daily living, and deterioration of functional and general health status. The unfavorable fracture sequelae combined with treatment disadvantages may also influence fragile balance of elderly functional capacities, thus speeding up disability and death, compared with general population. Authors of the multicenter, prospective study based on the cohort of the Study of Osteoporotic Fractures stated that occurrence of a wrist fracture increased the odds of suffering a clinically important functional decline and may play a role in the development of disability in older people [32]. Nevertheless, the exact underlying impact mechanism on mortality after distal radius fracture is unknown.

After subdividing into sex and age groups, we found excess mortality in men compared with women, even though men were younger at the time of the fracture. Similar observations were made by Rozental [28]. Potential explanations include men's greater functional decline and higher bone mineral loss resulting from the fact that osteoporosis is underdiagnosed and therefore undertreated, especially in this population [33]. Although the incidence of distal radius fractures is lower in men, the mortality is higher. This corresponding phenomenon is seen in other osteoporotic fractures. Remarkably, in the cohort of individuals with hip fractures from longitudinal Dubbo Osteoporosis Epidemiology Study (DOES), men have similarly greater excess mortality than women, even with statistically insignificant differences in comorbidities [34]. The abovementioned excess mortality was explained by the authors of this study, inter alia, by lifestyle factors. The dissimilarity in mortality between genders was, in our study, particularly pronounced after the 6^th^ month of follow-up. This may be connected with the end of outpatient's systematic care and aforementioned deepening of patient's malfunctioning. Similar explanation to observed excess men's mortality after 3^rd^ month, although in hip fracture population, was provided by Kannegaard [35]. Additionally, social issues may also be important [36]. Males tend to underreport acute health conditions [37, 38] and tend to disregard importance of preventive measures [39]. In context of osteoporotic fracture, the need for at least one-month DRF treatment and further rehabilitation coupled with restraining daily activity may be one of the reasons why mortality after distal radius fracture was significantly higher in men compared with women, whose compliance with those measures is better and so is the predicted functional outcome. Abovementioned suppositions should be examined in appropriate prospective study. Standard expected years of life lost (SEYLL) is a component of the disability adjusted life year (DALY) measure of disease burden [40]. SEYLLp index in our distal radius fracture cohort was higher in men than women: 1.6 years versus 0.6 years, respectively. It was, as expected, substantially lower than that reported after hip fracture in slightly older cohort of patients aged 55 and above: 5.9 years (males) versus 5.8 years (females). Interestingly, standard expected years of life lost in our DRF population was comparable with that of high-normal blood pressure men group (1.7 years) [41]. To sum up, distal radius fractures may play a role in the development of disability and additionally have influence on mortality, especially within male population.

We found that simple extra articular fractures such as AO type A and Frykman I may be predictors of mortality in a DRF cohort. To our knowledge, this is the first study ascertaining usefulness of common AO, Frykman, and Fernandez classification systems in predicting mortality in a DRF cohort. In the study, we analyzed three commonly used DRF classifications. Patients with simple, extra articular fractures were more likely to die than those with other types according to AO and Frykman altogether. The phenomenon can be explained by occurrence of simple fracture in fragile patients, with underlying osteoporosis. The ideal classification system should be a clear, concise, reproducible prognostic tool useful in determining outcomes, guiding treatment decision-making and predicting the possibility of complications, including mortality [42]. The debate on an ideal DRF classification system is ongoing [43]. Certainly, male sex and older age are very important factors affecting life expectancy in a distal radius fracture cohort. We suggest that these factors should be included systematically in DRF classification system. Thus, the new patient-accident-fracture (PAF) DRF classification may be a useful tool in targeting high-risk individuals with distal radius fracture [44].

The study has several limitations. Firstly, we do not have access to comorbidity data and preinjury functional status that may be confounding factors influencing mortality. Nevertheless, several studies prove that fracture may be an independent risk factor of excess mortality [14, 35]. In order to minimize the possible effect of unknown comorbidities we analyzed consecutive cohort of DRF fractures; we have controlled for age and sex and compared the study cohort with the background and standard populations. Secondly, another limitation, that should be considered, is that we have analyzed isolated distal radius fractures, with exclusion of multitrauma patients [28]. Multitrauma patients have higher mortality [45, 46], so probably when taking into account all individuals with DRF fractures, the mortality presented in the study was even underestimated and biased towards longer survival.

Throughout the world, public awareness of osteoporosis and the fragility fractures it causes is low [47]. Studies imply that second osteoporotic fracture, as well as higher mortality, may be prevented with appropriate interventions such as fall and frailty prevention, exercise, balance training with comprehensive rehabilitation program, diet, and antiosteoporotic drug implementation [48]. Preventive measures taken to date are reported to be insufficient [49]. Data suggest that after a diagnosis of DRF less than 20% of patients are referred for osteoporosis testing or treatment and even fewer receive fall prevention counseling [50]. Distal radius fractures occur earlier than hip or vertebral fractures and may signal the underlying problem, such as osteoporosis [51]. Likewise, as* fracture begets fracture*, patients with distal radius fractures, especially those aged 50 years and above, should be meticulously diagnosed and educated on possible underlying causes, risks of subsequent fractures, and necessity in widening diagnostics. FRAX calculation or BMD scan is reliable tool in diagnosing high-risk individuals [52]. Contact with the healthcare provider, on the occasion of distal radius fracture, should be an opportunity to case-find individuals who are at high risk of sustaining more severe fragility fractures in the future and systemically implement secondary prevention. The results of our study may help to target these high-risk individuals and thus better implement secondary prevention strategies.

To conclude, we found excess mortality in patients with distal radius fracture aged 50 years or above, especially in men and patients with simple extra articular fractures such as AO type I and Frykman I. The results of our study may help target high-risk individuals to whom particular attention should be paid in context of reducing mortality, which seems to be a challenge for healthcare professionals facing an epidemic of osteoporosis.

## Figures and Tables

**Table 1:** Cumulative mortality rates after 3, 6, and 9 months and 1 year of follow-up with standardized mortality ratios (SMRs) in the whole study group subdivided into sex and age subgroups. MR: mortality rate, DRF: distal radius fracture, 95% CI -95% confidence interval; SMR: standardized mortality ratio, p (mid): mid exact test.

Age group (years)	Follow up after DRF	All n=1308	Males n=256			Females n=1052		
		MR (%)	MR (%)	SMR (95%CI)	P (mid)	MR (%)	SMR (95%CI)	P (mid)

50-59	3m	0.2	0.4			0.4		
	6m	0.4	0.4			0.8		
	9m	0.9	0.9			0.8		
	1yr	0.9	1.0	0.9 (0.1-4.3)	0.63	0.8	1.9 (0.1-9.2)	0.52

60-69	3m	0.1	0.7			0.2		
	6m	0.3	0.7			0.3		
	9m	1.1	2.9			0.7		
	1yr	1.7	4.4	1.8 (0.5 -4.9)	0.32	1.0	1.0 (0.0-1.6)	0.24

>70	3m	2.1	3.6			1.9		
	6m	2.9	8.3			2.2		
	9m	5.2	15.5			3.8		
	1yr	6.5	17.9	2.4 (1.4-3.9)	0.002	5.1	0.9 (0.6-1.2)	0.46

Total 50+	3m	1.2	1.2			1.1		
	6m	1.8	2.7			1.5		
	9m	3.3	6.3			2.6		
	1yr	4.5	7.4	2.5 (1.5-3.8)	<0.001	3.4	1.5 (1.0-2.0)	0.05

## Data Availability

The mortality data used to support the findings of this study are included within the article and are fully available from the corresponding author upon request.
